# Endothelial Function in Patients with Multiple Sclerosis: The Role of GLP-1 Agonists, Lipoprotein Subfractions, and Redox Balance

**DOI:** 10.3390/ijms241311162

**Published:** 2023-07-06

**Authors:** Miroslava Hardonova, Pavel Siarnik, Monika Sivakova, Bianka Sucha, Adela Penesova, Zofia Radikova, Andrea Havranova, Richard Imrich, Miroslav Vlcek, Ingrid Zitnanova, Georgi Krastev, Maria Kiacikova, Branislav Kollar, Peter Turcani

**Affiliations:** 1 1st Department of Neurology, Faculty of Medicine, Comenius University, 813 69 Bratislava, Slovakia; 2 Institute of Clinical and Translational Research, Biomedical Research Center, Slovak Academy of Sciences, 845 05 Bratislava, Slovakia; 3 Institute of Medical Chemistry, Biochemistry and Clinical Biochemistry, Faculty of Medicine, Comenius University, 811 08 Bratislava, Slovakia; 4 Department of Neurology, Faculty Hospital, 917 75 Trnava, Slovakia; 5 Department of Neurology, Faculty Hospital, 911 01 Trencin, Slovakia

**Keywords:** multiple sclerosis, GLP-1 agonists, lipoprotein subfractions, redox balance, endothelial dysfunction

## Abstract

Introduction: Epidemiological studies have suggested an increased vascular risk in patients with multiple sclerosis (MS). There is increasing evidence of the beneficial effects of GLP-1 agonists (GLP-1a) in preventing vascular complications and slowing the progression of neurodegeneration. Our objective was to explore the changes in the endothelial function of MS patients after 12 months of GLP-1a therapy. We also explored the role of lipoprotein subfractions and the antioxidant capacity of plasma. Methods: MS patients were enrolled in a prospective, unicentric study. GLP-1a (dulaglutide) was administered to 13 patients. The control population consisted of 12 subjects. Endothelial function was determined by peripheral arterial tonometry and expressed as reperfusion hyperemia index (RHI). Trolox equivalent antioxidant capacity (TEAC) was used to assess the total antioxidant capacity of the plasma. The levels of lipoprotein subfractions were evaluated. Results: The GLP-1a group did not have a significant change in their RHIs after 12 months (2.1 ± 0.6 vs. 2.1 ± 0.7; *p* = 0.807). However, a significant increase in their TEACs was observed (4.1 ± 1.4 vs. 5.2 ± 0.5 mmol/L, *p* = 0.010). On the contrary, the subjects in the control group had a significant worsening of their RHIs (2.1 ± 0.5 vs. 1.8 ± 0.6; *p* = 0.030), without significant changes in their TEACs. Except for a significant decrease in very-low-density lipoprotein (VLDL) (30.8 ± 10.2 vs. 22.6 ± 8.3 mg/dL, *p* = 0.043), no other significant changes in the variables were observed in the control group. VLDL levels (beta = −0.637, *p* = 0.001), the use of GLP-1a therapy (beta = 0.560, *p* = 0.003), and small LDL (beta = 0.339, *p* = 0.043) were the only significant variables in the model that predicted the follow-up RHI. Conclusion: Our results suggest that the application of additional GLP-1a therapy may have atheroprotective and antioxidant effects in MS patients with high MS activity and thus may prospectively mitigate their vascular risk. However, the lipoprotein profile may also play an important role in the atherogenic risk of MS subjects.

## 1. Introduction

Multiple sclerosis (MS) is a chronic disease of the central nervous system (CNS) that leads to demyelination and neurodegeneration. It usually affects younger adults with a certain genetic predisposition [1]. In a previous ongoing study at our institutions, we observed signs of insulin resistance (IR) and postprandial hyperinsulinemia in patients with newly diagnosed MS [2,3]. Studies exploring the role of IR in other chronic inflammatory diseases have suggested that chronic inflammation or reduced physical activity do not belong to the only key pathomechanisms involved in the development of IR [4,5]. Some common mechanisms, such as mitochondrial dysfunction, may be involved in the pathogeneses of insulin resistance and neurodegeneration. Mitochondrial dysfunction can lead to insufficient energy production necessary to maintaining normal membrane potential [6]. Consequently, neurons with impaired membrane potential are susceptible to damage by oxidative stress [7]. Reactive oxygen species (ROS) can also originate from activated microglia, which are involved in immune protection [8]. Despite controversial findings in subjects with newly diagnosed MS, prolonged oxidative stress in patients with MS could be associated with subsequent atherogenesis [5]. The elevation of some markers of endothelial dysfunction has also been observed in MS patients [9,10]. Endothelial dysfunction is considered to be one of the most important initial steps in the atherogenesis process [11]. The findings of epidemiological studies have suggested that MS patients have a higher incidence of cardiovascular and cerebrovascular diseases. This increased vascular morbidity is not fully dependent on traditional vascular risk factors [12]. Although atherogenic dyslipidemia caused by chronic systemic inflammation in MS patients could be one of the important contributors to atherogenesis, the exact mechanism underlying this increased vascular morbidity remains unknown [13]. Other findings have suggested that changes in lipoprotein subclasses may be associated with insulin resistance in MS subjects [14]. Endothelial dysfunction can initiate the development of atherosclerotic changes and thus the development of vascular complications in MS patients [15]. There is increasing evidence for the beneficial effects of GLP-1 agonists (GLP-1as) in preventing vascular complications and slowing neurodegeneration [16]. GLP-1as belong to the so-called “new antidiabetic drugs” and are used worldwide for the treatment of type 2 diabetes mellitus and obesity [17]. GLP-1 is an incretin whose secretion by L cells in the ileum is stimulated by oral food intake, especially carbohydrates and fats. It acts through a specific GLP-1 receptor (GLP-1R) and its main functions include the stimulation of glucose-dependent insulin secretion, the alleviation of insulin desensitization, the inhibition of glucagon washout, and the slowing of gastric emptying. GLP-1 is also produced by neurons in the nucleus tractus solitarii of the brainstem, where it acts as a neurotransmitter. Therefore, in addition to its aforementioned “incretin effect”, GLP-1a has neuroprotective, neurotrophic, and anti-inflammatory effects [18,19]. In addition, they reduce oxidative-stress-induced apoptosis in the progenitor cells of the target organ, thus decreasing ROS production [19,20]. These effects are relevant due to the complications and increased vascular risk in patients with MS. The current study aimed to explore the changes in the endothelial function parameters of MS patients after 12 months of GLP-1as therapy and in a control group of MS patients without GLP-1a therapy. We also aimed to explore the follow-up predictors of endothelial function in these MS subjects, including their lipoprotein subfractions and plasma antioxidant capacity.

## 2. Results

GLP-1a therapy was administered to 13 patients (6 women and 7 men, age: 44.9 ± 7.7 years, BMI: 27.8 ± 5.1 kg/m^2^, smoking habit: 2 (15.4%), arterial hypertension: 2 (15.4%), and statin use: 1 (7.7%)). The control population without additional GLP-1a therapy consisted of 12 subjects (6 women and 6 men, age: 35.8 ± 5.4 years, BMI: 23.9 ± 3.9 kg/m^2^, smoking habit: 1 (8.3%), arterial hypertension: 0 (0%), and statin use: 0 (0%)). The other recorded baseline and follow-up demographic characteristics and vascular risk factors are included in Table 1. The patients in the GLP-1a group were significantly older and had significantly higher BMIs. Between the groups, there was no significant difference in gender, disease severity, disease duration, and vascular risk factors, including arterial hypertension, smoking habit, and statin use. During our examinations, all the patients were in remission of MS.

A baseline and follow-up comparison of the other clinical characteristics between the GLP-1a group and control group is included in Table 2 and Table 3.

**Figure 1:**
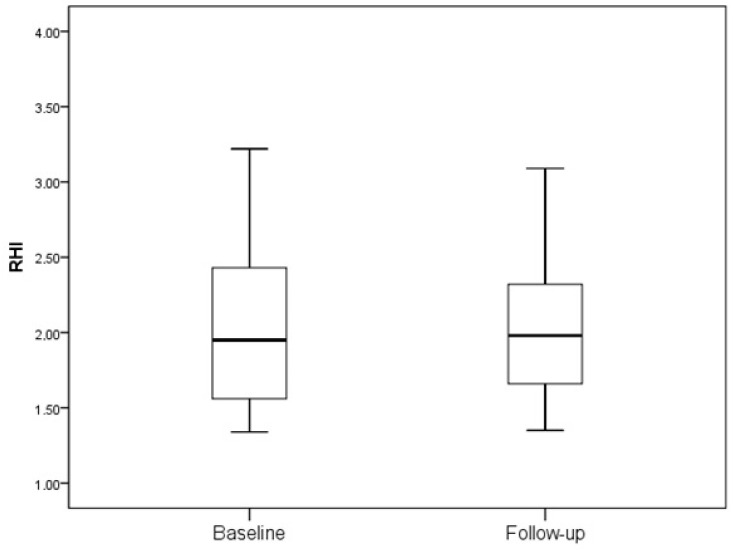
RHI in subjects receiving GLP-1a (2.1 ± 0.6 vs. 2.1 ± 0.7; *p* = 0.807).

**Figure 2:**
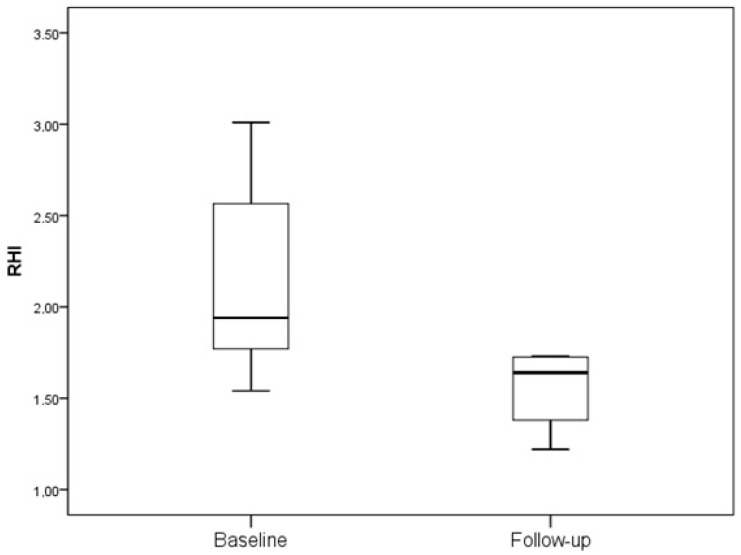
RHI in subjects who did not receive GLP-1a (2.1 ± 0.5 vs. 1.8 ± 0.6; *p* = 0.030).

In both groups, during a 12-month period, no change in the frequency of vascular risk factors, including smoking, arterial hypertension, or statin use, was recorded. There was no significant change in RHIs (2.1 ± 0.6 vs. 2.1 ± 0.7; *p* = 0.807) after 12 months of GLP-1a treatment in the subjects treated with GLP-1a (Figure 1). In contrast, in the control group, a significant worsening of their RHIs was observed (2.1 ± 0.5 vs. 1.8 ± 0.6; *p* = 0.030) (Figure 2).

Similarly, no significant change in BMIs was observed in the GLP-1a therapy group (27.8 ± 5.1 to 27.1 ± 4.8 kg/m^2^, *p* = 0.705) and control group (23.9 ± 3.9 to 24.3 ± 4.2 kg/m^2^, *p* = 0.795). In the GLP-1a therapy group, no significant changes in any of their lipoprotein levels were observed. However, a significant increase in TEACs was observed over a 12-month period in this group (4.1 ± 1.4 vs. 5.2 ± 0.5 mmol/L, *p* = 0.010). In the control group, except for a significant decrease in VLDL (30.8 ± 10.2 vs. 22.6 ± 8.3 mg/dL, *p* = 0.043), no significant changes in any other lipoprotein levels or TEACs were observed (Table 4 and Table 5).

**Figure 3:**
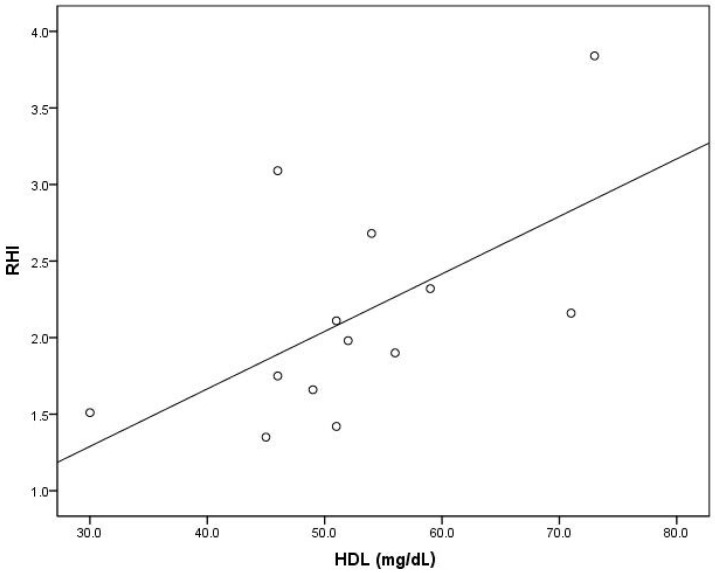
Association of the RHI with HDL in subjects receiving GLP-1a (circles describe association in particular subjects).

**Figure 4:**
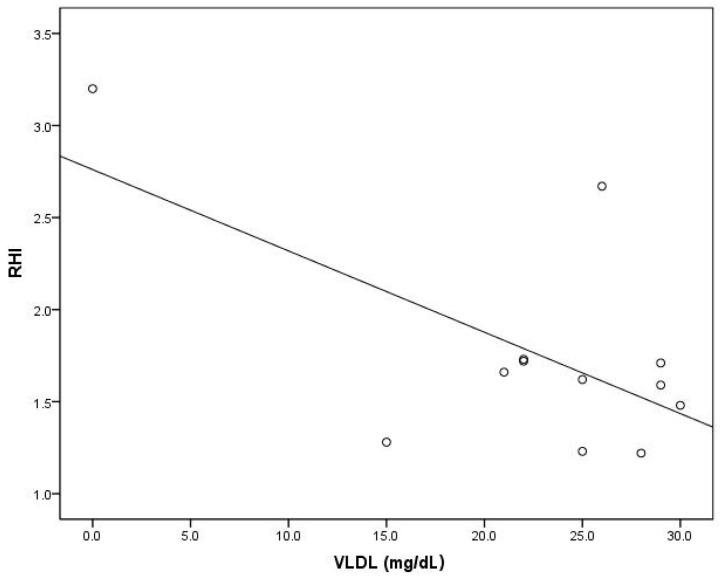
Association of the RHI with VLDL in the control group (circles describe association in particular subjects).

In the model that predicts the follow-up RHIs, VLDL levels (beta = −0.637, *p* = 0.001), the use of GLP-1a therapy (beta = 0.560, *p* = 0.003), and small LDL (beta = 0.339, *p* = 0.043) were the only significant contributors in the entire study population. When each group was considered separately, HDL level (beta = 0.585, *p* = 0.036) was the only significant contributor in the model predicting the follow-up RHIs in the GLP-1a group, and VLDL level (beta = −0.621, *p* = 0.031) was the only significant contributor in the model predicting the follow-up RHIs in the control group (Table 6, Figure 3 and Figure 4).

## 3. Discussion

Our results suggest a significant deterioration of endothelial function over 12 months in a population of relapsing-remitting MS patients with high disease activity without additional GLP-1a therapy. Additionally, a significant decrease in VLDL levels was found in this group. In these subjects, we were unable to find any significant changes in the other parameters observed. In contrast, there was no significant change in the endothelial function in the population with additional GLP-1a treatment. Additionally, a significant increase in TEACs was found in this group. VLDL levels (beta = −0.637, *p* = 0.001), GLP-1a therapy (beta = 0.560, *p* = 0.003), and small LDL (beta = 0.339, *p* = 0.043) were the only significant variables in the model that predicted the follow-up RHI. No significant role of TEAC was detected. These findings suggest a possible antioxidant and atheroprotective role of GLP-1a.

According to previous studies, a higher incidence of vascular disease, as well as endothelial dysfunction, is known in MS patients. Endothelial dysfunction could play a key role in the development of vascular complications in these patients [9,10,12,15]. Our findings are consistent with the above facts. Currently, GLP-1a is used as a new class of drugs for the treatment of type 2 diabetes mellitus, but it has also been shown to have a protective effect on the cardiovascular system [21]. In several studies, the beneficial effects of GLP-1a have been observed on cardiovascular complications and neurodegenerative disorders of the central nervous system, suggesting their therapeutic potential beyond improving diabetes complications [22]. Their effect on known cardiovascular risk factors such as hyperglycemia, a higher BMI, increased blood pressure, and unfavorable lipoprotein levels is also considered. GLP-1a also affects several biological processes in the blood vessels. An effect on the low-grade inflammatory process and an improvement in plaque stability has been described. Similarly, a slowing effect on the process of the development, progression, and rupture of atherosclerotic plaques is suspected [23]. The impact on the progression of the intima-media thickness (IMT) of the common carotid artery suggests a role of the GLP-1a in the slowing of the atherosclerosis process [24]. An additional role may also be played in reducing inflammatory cytokines and ROS production, which can contribute to a reduction in atherosclerosis progression [25]. As a result of reduced ROS production, GLP-1as also have a protective effect against oxidative damage [26], which may reduce the risk of subsequent microvascular injury [27]. In addition, some types of GLP-1as can enhance acetylcholine-induced vasodilation [28]. In another study, GLP-1as also improved the NO synthase activity in human endothelial cells [29]. Our findings are consistent with these previous findings and suggest a possible atheroprotective and antioxidant role for GLP-1as in MS subjects. The use of GLP-1a therapy (beta = 0.560, *p* = 0.003) belongs, among significant contributors, to the model predicting the follow-up RHI. We are not aware of any similar findings in the MS population so far. Our findings also suggest a possible role of the lipoprotein profile in the atherogenic risk of MS subjects. Our results in the entire study population support the well-known evidence of the atherogenic properties of VLDL [30]. However, despite the borderline significance, our results suggest an atheroprotective role of small LDL (beta = 0.339, *p* = 0.043). This finding is in contrast to previous studies [31,32,33,34]. When each study subgroup was considered separately, the HDL level was the only significant contributor in the model predicting the follow-up RHIs in the GLP-1a group, and the VLDL level was the only significant contributor in the model predicting the follow-up RHIs in the control group. These findings suggest well-known atheroprotective properties of HDL and well-known atherogenic properties of VLDL [30]. The elucidation of the atherogenic/atheroprotective properties of lipoprotein subfractions in neurologic diseases requires further extensive research.

Most likely, GLP-1as have antiatherosclerotic effects that appear to be partially independent of their apparent improvement in the risk factors that accompany such treatment [35,36]. There are also known so-called pleiotropic effects of GLP-1a, which include a cytoprotective effect in various cell types, including cardiomyocytes [37]. Of particular importance is the role of GLP-1 in oxidative pathways. The interaction of GLP-1 with its receptors induces antioxidant effects by amplifying the cyclic adenosine monophosphate (cAMP), phosphoinositide 3-kinase (PI3K), and protein kinase C (PKC) pathways. The result is an increased expression of antioxidant enzymes and an increased activation of the nuclear factor erythroid-related factor 2 (Nrf2-ARE) pathway [38,39]. Nrf2 is a transcription factor which increases the expressions of antioxidants such as nicotinamide adenine dinucleotide phosphate [NAD(P)H] dehydrogenase, superoxide dismutase, and glutathione peroxidase 29, with a consequent decrease in reactive oxygen species [40]. An increased Nrf2 expression due to GLP-1a has a cardioprotective effect that counteracts oxidative stress. Furthermore, GLP-1 reduces oxidative-stress-induced apoptosis in the progenitor cells of target organs. This mechanism is mediated by the activation of its receptor and a subsequent increase in protein kinase A (PKA) activity, with a subsequent inhibition of the signaling cascade of the MKK4/MKK7/JNK (mitogen-activated protein kinase 4/7, N-terminal kinase) signaling cascade that mediates apoptosis [41].

There is another possible mechanism by which GLP-1as lead to a reduced risk of atherogenesis. GLP-1as reduce hunger mediated by the receptors expressed in the central nervous system and thus cause a modest weight loss [36]. This weight loss, in addition to slowing down the atheroma formation and improving the endothelial and platelet function, may have a protective effect on thromboembolic events in patients with an increased cardiovascular risk. However, no significant reduction in BMI was observed in the current study.

Several limitations of the current study should be noted. The most significant limitation was the small sample size. Our results need to be validated in larger prospective studies. The design of the current work does not allow us to elucidate in detail the mechanisms by which the potential atheroprotective effect of GLP-1a is mediated. However, we would like to highlight that the current paper is a brief report presenting the preliminary results of ongoing research. The enrollment of the patients was slowed down by the COVID-19 pandemic, and some of the patients could have been discouraged by the subcutaneous form of GLP-1a that was used in the current study. The enrollment process is ongoing. We suppose that the use of newly available peroral GLP-1a agents may help to expand the number of enrolled subjects. The ongoing research aims to elucidate the possible impact of GLP-1a on multiple measures of neurodegeneration (including magnetic resonance volumetry and neurofilament light chain levels), glucose metabolism, and oxidative stress.

Future works should examine in more detail the influence of GLP-1as on a wide range of traditional (glucose metabolism, blood pressure regulation, anthropometric parameters, dietary habits, and lifestyle) and nontraditional vascular factors (sleep disturbances and gut microbiome, etc.). According to the findings of the current study, interventions influencing the lipoprotein profiles in MS subjects should have the highest priority.

We have to admit one more limitation of the current study. Despite no significant differences between the GLP-1a group and control group in gender, disease severity, disease duration, and frequency of vascular risk factors, the subjects in the GLP-1a group were significantly older and had significantly higher BMIs. This partly limits the interpretation of the results in the entire study population. However, the results in the particular subgroups should be more reliable, because there was no significant change in the demographic characteristics within the GLP-1a and control subgroup during a 12-month period. A better matching of the GLP-1a group and control group is highly warranted in ongoing research.

## 4. Materials and Methods

### 4.1. A Study Population

The study was carried out at the 1st Department of Neurology, Faculty of Medicine, Comenius University, Bratislava, Slovakia. The study was approved by the local ethics committee under the reference VV-2018-R-EK. All the patients provided their informed consent. Patients with a confirmed diagnosis of a relapsing-remitting form of MS, with a duration of the disease of at least 1 year, who were on natalizumab therapy at the time of examination, were enrolled in a prospective, unicentric study. Only patients with an Expanded Disability Status Scale (EDSS) of <5 were enrolled. The main exclusion criteria included having diabetes mellitus, being on oral antidiabetic drugs or insulin, or suffering from other major chronic diseases such as cancer, followed by the long-term use of medications such as glucocorticoids or antidepressants. Additional exclusion criteria included drug abuse, alcohol abuse, and a known hypersensitivity to the GLP-1a administered. The exclusion criteria for women included pregnancy or breastfeeding at the time of inclusion or during the study. The patients were then divided into 2 groups. One group received GLP-1a therapy (dulaglutide 0.75 mg sc, once a week) for 12 months, and the second group consisted of individuals without additional GLP-1a therapy. The patients were examined twice: they underwent an initial examination before their inclusion in the study and a second examination after 12 months. Inclusion in the study was preceded by a detailed explanation of the study process, potential risks, and adverse effects. Each participant signed an informed consent to participate in the study. The examinations were performed at 8:00 a.m. in fasting conditions. The patients were advised to avoid significant physical exertion on the previous day and in the morning prior to the examination.

### 4.2. Evaluation of Endothelial Function

Endothelial function was assessed by peripheral arterial tonometry (EndoPAT 2000, Itamar Medical, Caesarea, Israel) and expressed as reperfusion hyperemia index (RHI). This is a noninvasive examination based on the determination of nitric-oxide-dependent vasodilation. It is determined by the change in the pulse wave amplitude in reperfusion hyperemia (post-occlusive hyperemia after occlusion of the brachial artery by the manometer cuff) compared to the resting pulse wave amplitude.

The measurement was calculated using the automated algorithm (software Version 3.1.2) provided with the device. The measurements were made according to the developer’s instructions, as previously described [11]. The patients were in the supine position for a minimum of 20 min before the measurement in a quiet room and asked to remain completely still and quiet throughout the measurement period. Each recording consisted of 5 min of baseline measurement, 5 min of occlusion measurement, and 5 min of post-occlusion measurement (hyperemic period). Brachial artery occlusion was performed on the arm of the non-dominant limb. The occlusion pressure was at least 60 mmHg higher than the systolic blood pressure. An RHI rate of ≤1.67 was considered to be endothelial dysfunction [42].

### 4.3. Lipoprotein Levels

Blood plasma samples were obtained at 8:00 am in fasting conditions. Samples with EDTA (ethylenediaminetetraacetic acid) were collected. The enzymatic method (Roche Diagnostics, Mannheim, Germany) was used to determine the levels of total cholesterol (TC), low-density lipoprotein cholesterol (LDL), and high-density lipoprotein cholesterol (HDL). Furthermore, the Lipoprint system (Quantimetrix Corp., Redondo Beach, CA, USA), using polyacrylamide gel electrophoresis, was used for the quantitative analysis of the lipoprotein families and lipoprotein subfractions, including very-low-density lipoprotein (VLDL), intermediate-density lipoprotein (IDL), large LDL subfractions (1–2), small dense LDL subfractions (3–7), large HDL subfractions (1–3), intermediate HDL subfractions (4–7), and small dense HDL subfractions (8–10) [43]. Large LDL subfractions and large HDL subfractions are considered to be atheroprotective. On the other hand, small dense LDL subfractions and small dense HDL subfractions are considered to be atherogenic. The atherogenic/atheroprotective role of intermediate HDL subfractions remains controversial [31,32].

### 4.4. Assessment of Antioxidant Capacity

The collected blood was centrifuged for 5 min at 1200× *g* at 4 °C. The serum samples were stored in aliquots at −70 °C until they were analyzed for their total antioxidant capacity. The Trolox equivalent antioxidant capacity (TEAC) assay was used for the assessment of the total antioxidant capacity [44].

### 4.5. Statistical Analysis

The statistical analysis was performed using the IBM SPSS statistics software package version 25. The categorical variables were expressed as numbers and percentages (%). The continuous variables were expressed as mean ± standard deviation. The Student’s *t* test was used to compare the assessed values at the time of enrollment and after 12 months. A stepwise multiple linear regression analysis was used to create the RHI prediction model. We chose a model with the highest number of significant predictors. The dependent variable was the RHI, the independent variables in the model were the BMI, TEAC, lipoprotein levels (TC, VLDL, IDL, LDL, HDL, large HDL, intermediate HDL, small HDL large LDL, and small LDL), and GLP-1a therapy status (in follow-up). The variables in each model were evaluated for multicollinearity, and variance inflation factors (VIF) of ≤5 were indicative of multicollinearity. A *p* value of <0.05 was considered to be statistically significant.

## 5. Conclusions

Our results suggest that the application of additional therapy with GLP-1as may have atheroprotective and antioxidant effects in MS patients with high MS activity and thus may prospectively mitigate their vascular risk. However, the lipoprotein profile may also play an important role in the atherogenic risk of MS subjects.

## Figures and Tables

**Table 1:** Demographic characteristics of GLP-1a group and control group.

	Baseline			Follow-Up		
	GLP-1a Group	Control Group	*p*	GLP-1a Group	Control Group	*p*
Age (years)	44.9 ± 7.7	35.8 ± 5.4	0.002 **	45.9 ± 7.7	36.8 ± 5.4	0.002 **
Female/male	6/7 (46.2/53.8%)	6/6 (50/50%)	0.848	6/7 (46.2/53.8%)	6/6 (50/50%)	0.848
Disease duration (months)	144.0 ± 44.6	136.0 ± 40.1	0.643	156.0 ± 44.6	148.0 ± 40.1	0.643
EDSS	4.0, 2.25 (1.0–4.5)	1.5, 3.5 (0–4.5)	0.077	4.0, 2.25 (1.0–4.5)	1.5, 3.5 (0–4.5)	0.077
BMI (kg/m^2^)	27.8 ± 5.1	23.9 ± 3.9	0.040 *	27.1 ± 4.8	24.3 ± 4.2	0.135
Arterial hypertension	2 (15.4%)	0 (0%)	0.157	2 (15.4%)	0 (0%)	0.157
Statin use	1 (7.7%)	0 (0%)	0.327	1 (7.7%)	0 (0%)	0.327
Smoking	2 (15.4%)	1 (8.3%)	0.588	2 (15.4%)	1 (8.3%)	0.588

*: *p* ˂ 0.05; **: *p* ˂ 0.01.

**Table 2:** Baseline comparison between GLP-1a group and control group.

	GLP-1a Group	Control Group	*p*
BMI (kg/m^2^)	27.8 ± 5.1	23.9 ± 3.9	0.040 *
RHI	2.1 ± 0.6	2.1 ± 0.5	0.792
TC (mg/dL)	200.6 ± 39.7	168.5 ± 29.2	0.032 *
LDL (mg/dL)	112.3 ± 34.3	84.2 ± 16.6	0.017 *
HDL (mg/dL)	53.2 ± 11.9	53.3 ± 16.6	0.986
VLDL (mg/dL)	34.9 ± 10.3	30.8 ± 10.2	0.329
IDL (mg/dL)	45.8 ± 11.9	37.8 ± 8.5	0.067
Small LDL (mg/dL)	1.0, 5.0 (0–27.0)	0, 2.0 (0–4.0)	0.376
Large LDL (mg/dL)	58.0, 30.0 (31.0–104.0)	45.0, 18.5 (26.0–66.0)	0.035 *
Small HDL (mg/dL)	8.0, 6.0 (0.0–14.0)	6.5, 4.0 (4.0–10.0)	0.376
Intermediate HDL (mg/dL)	29.0, 9.0 (22.0–42.0)	29.8, 8.0 (24.0–44.0)	0.574
Large HDL (mg/dL)	13.0, 15.0 (5.0–25.0)	12.5, 19.0 (5.0–37.0)	0.769
TEAC (mmol/L)	4.1 ± 1.4	4.3 ± 1.4	0.704

*: *p* ˂ 0.05.

**Table 3:** Follow-up comparison between GLP-1a group and control group.

	GLP-1a Group	Control Group	*p*
BMI (kg/m^2^)	27.1 ± 4.8	24.3 ± 4.2	0.135
RHI	2.1 ± 0.7	1.8 ± 0.6	0.166
TC (mg/dL)	184.8 ± 22.3	157.7 ± 23.5	0.007 **
LDL (mg/dL)	103.0 ± 18.4	84.3 ± 19.2	0.020 *
HDL (mg/dL)	52.5 ± 11.1	50.6 ± 14.5	0.707
VLDL (mg/dL)	29.2 ± 11.9	22.6 ± 8.3	0.130
IDL (mg/dL)	43.4 ± 14.5	34.6 ± 6.6	0.068
Small LDL (mg/dL)	0, 1.5 (0–12.0)	2.0, 3.5 (0–15.0)	0.168
Large LDL (mg/dL)	59.0, 16.0 (44.0–79.0)	44.5, 20.0 (26.0–77.0)	0.040 *
Small HDL (mg/dL)	8.0, 6.0 (5.0–19.0)	7.0, 4.0 (2.0–11.0)	0.728
Intermediate HDL (mg/dL)	28.0, 6.0 (18.0–40.0)	29.0, 11.0 (18.0–37.0)	0.894
Large HDL (mg/dL)	16.0, 11.0 (6.0–28.0)	13.0, 15.0 (3.0–35.0)	0.810
TEAC (mmol/L)	5.2 ± 0.5	4.7 ± 1.2	0.172

*: *p* ˂ 0.05; **: *p* ˂ 0.01.

**Table 4:** Changes in baseline measures in the GLP-1a group.

	Baseline	Follow-Up	*p*
BMI (kg/m^2^)	27.8 ± 5.1	27.1 ± 4.8	0.705
RHI	2.1 ± 0.6	2.1 ± 0.7	0.807
TC (mg/dL)	200.6 ± 39.7	184.8 ± 22.3	0.224
LDL (mg/dL)	112.3 ± 34.3	103.0 ± 18.4	0.397
HDL (mg/dL)	53.2 ± 11.9	52.5 ± 11.1	0.879
VLDL (mg/dL)	34.9 ± 10.3	29.2 ± 11.9	0.197
IDL (mg/dL)	45.8 ± 11.9	43.4 ± 14.5	0.641
Small LDL (mg/dL)	1.0, 5.0 (0–27.0)	0, 1.5 (0–12.0)	0.362
Large LDL (mg/dL)	58.0, 30.0 (31.0–104.0)	59.0, 16.0 (44.0–79.0)	0.762
Small HDL (mg/dL)	8.0, 6.0 (0.0–14.0)	8.0, 6.0 (5.0–19.0)	0.840
Intermediate HDL (mg/dL)	29.0, 9.0 (22.0–42.0)	28.0, 6.0 (18.0–40.0)	0.448
Large HDL (mg/dL)	13.0, 15.0 (5.0–25.0)	16.0, 11.0 (6.0–28.0)	0.920
TEAC (mmol/L)	4.1 ± 1.4	5.2 ± 0.5	0.010 *

*: *p* ˂ 0.05.

**Table 5:** Changes in baseline measures in the control group.

	Baseline	Follow-Up	*p*
BMI (kg/m^2^)	23.9 ± 3.9	24.3 ± 4.2	0.795
RHI	2.1 ± 0.5	1.8 ± 0.6	0.030 *
TC (mg/dL)	168.5 ± 29.2	157.7 ± 23.5	0.332
LDL (mg/dL)	84.2 ± 16.6	84.3 ± 19.2	0.991
HDL (mg/dL)	53.3 ± 16.6	50.6 ± 14.5	0.669
VLDL (mg/dL)	30.8 ± 10.2	22.6 ± 8.3	0.043 *
IDL (mg/dL)	37.8 ± 8.5	34.6 ± 6.6	0.306
Small LDL (mg/dL)	0, 2.0 (0–4.0)	2.0, 3.5 (0–15.0)	0.178
Large LDL (mg/dL)	45.0, 18.5 (26.0–66.0)	44.5, 20.0 (26.0–77.0)	0.843
Small HDL (mg/dL)	6.5, 4.0 (4.0–10.0)	7.0, 4.0 (2.0–11.0)	0.443
Intermediate HDL (mg/dL)	29.8, 8.0 (24.0–44.0)	29.0, 11.0 (18.0–37.0)	0.671
Large HDL (mg/dL)	12.5, 19.0 (5.0–37.0)	13.0, 15.0 (3.0–35.0)	0.887
TEAC (mmol/L)	4.3 ± 1.4	4.7 ± 1.2	0.441

*: *p* ˂ 0.05.

**Table 6:** Association of follow-up measures with RHI.

	All Subjects		GLP-1a Group		Control Group	
	Linear Regression		Linear Regression		Linear Regression	
	beta	*p*	beta	*p*	beta	*p*
BMI (kg/m^2^)	−0.091	0.598	0.021	0.941	0.002	0.995
TC	0.156	0.405	−0.103	0.702	0.125	0.657
LDL	0.074	0.687	0.050	0.949	0.119	0.706
HDL	0.181	0.300	0.585	0.036 *	0.085	0.766
VLDL	−0.637	0.001 *	−0.373	0.214	−0.621	0.031 *
IDL	0.020	0.912	0.007	0.981	0.116	0.677
Small LDL	0.339	0.043 *	0.073	0.782	0.262	0.343
Large LDL	0.083	0.657	0.050	0.867	−0.036	0.920
Small HDL	0.167	0.389	−0.010	0.969	0.121	0.655
Intermediate HDL	0.220	0.180	0.136	0.816	0.137	0.612
Large HDL	0.073	0.705	−0.045	0.912	−0.006	0.986
TEAC	0.175	0.310	0.338	0.187	0.143	0.604
GLP-1a use	0.560	0.003 *	-	-	-	-

*: *p* ˂ 0.05.

## Data Availability

The data presented in this study are available on request from the corresponding author.

## Abbreviations

MS	Multiple sclerosis
CNS	Central nervous system
ROS	Reactive oxygen species
GLP-1	Glucagon like peptide 1
GLP-1as	Glucagon like peptide 1 agonists
GLP-1R	Glucagon like peptide 1 receptor
EDSS	Expanded Disability Status Scale
RHI	Reperfusion hyperemia index
IMT	Intima-media complex
cAMP	Cyclic adenosine triphosphate
PI3K	Phosphoinositide 3-kinase
PKA, PKC	Protein kinase A, C
Nrf2-ARE	Nuclear factor erythroid-related factor 2
NAD(P)H	Nicotinamide adenine dinucleotide phosphate
MKK4/MKK7	Mitogen-activated protein kinase kinase 4/7
JNK	c-Jun N-terminal kinase
NO synthase	Nitric oxide synthase

## References

[1] Rostami A., Ciric B. (2013). Role of Th17 cells in the pathogenesis of CNS inflammatory demyelination. J. Neurol. Sci..

[2] Penesova A., Vlcek M., Imrich R., Vernerova L., Marko A., Meskova M., Grunnerova L., Turcani P., Jezova D., Kollar B. (2015). Hyperinsulinemia in newly diagnosed patients with multiple sclerosis. Metab. Brain Dis..

[3] Penesová A., Dean Z., Kollár B., Havranová A., Imrich R., Vlček M., Rádiková Ž. (2018). Nutritional intervention as an essential part of multiple sclerosis treatment?. Physiol. Res..

[4] Penesova A., Rovensky J., Zlnay M., Dedik L., Radikova Z., Koska J., Vigas M., Imrich R. (2005). Attenuated insulin response and normal insulin sensitivity in lean patients with ankylosing spondylitis. Int. J. Clin. Pharmacol. Res..

[5] Sivakova M., Siarnik P., Filippi P., Vlcek M., Imrich R., Turcani P., Zitnanova I., Penesova A., Radikova Z., Kollar B. (2019). Oxidative stress in patients with newly diagnosed multiple sclerosis: Any association with subclinical atherosclerosis?. Neuroendocrinol. Lett..

[6] Vlcek M., Penesova A., Imrich R., Meskova M., Mravcova M., Grunnerova L., Garafova A., Sivakova M., Turcani P., Kollar B. (2018). Autonomic Nervous System Response to Stressors in Newly Diagnosed Patients with Multiple Sclerosis. Cell. Mol. Neurobiol..

[7] Prokopova B., Hlavacova N., Vlcek M., Penesova A., Grunnerova L., Garafova A., Turcani P., Kollar B., Jezova D. (2017). Early cognitive impairment along with decreased stress-induced BDNF in male and female patients with newly diagnosed multiple sclerosis. J. Neuroimmunol..

[8] Bhatti J.S., Bhatti G.K., Reddy P.H. (2017). Mitochondrial dysfunction and oxidative stress in metabolic disorders—A step towards mitochondria based therapeutic strategies. Biochim. Biophys. Acta Mol. Basis Dis..

[9] Minagar A., Jy W., Jimenez J.J., Sheremata W.A., Mauro L.M., Mao W.W., Horstman L.L., Ahn Y.S. (2001). Elevated plasma endothelial microparticles in multiple sclerosis. Neurology.

[10] Keményová P., Siarnik P., Sutovský S., Blaho A., Turcáni P., Kollár B. (2015). Impairment of endothelial function in patients with multiple sclerosis. Neuroendocrinol. Lett..

[11] Siarnik P., Carnicka Z., Krizova L., Wagnerova H., Sutovsky S., Klobucnikova K., Kollar B., Turcani P., Sykora M. (2014). Predictors of impaired endothelial function in obstructive sleep apnea syndrome. Neuroendocrinol. Lett..

[12] Marrie R.A., Reider N., Cohen J., Stuve O., Trojano M., Cutter G., Reingold S., Sorensen P.S. (2015). A systematic review of the incidence and prevalence of cardiac, cerebrovascular, and peripheral vascular disease in multiple sclerosis. Mult. Scler..

[13] Rádiková Ž., Penesová A., Vlček M., Havranová A., Siváková M., Šiarnik P., Žitňanová I., Imrich R., Turčáni P., Kollár B. (2020). Lipoprotein profiling in early multiple sclerosis patients: Effect of chronic inflammation?. Lipids Health Dis..

[14] Radikova Z., Penesova A., Vlcek M., Havranova A., Sivakova M., Siarnik P., Zitnanova I., Imrich R., Kollar B., Turcani P. (2018). LDL and HDL lipoprotein subfractions in multiple sclerosis patients with decreased insulin sensitivity. Endocr. Regul..

[15] Christiansen C.F., Christensen S., Farkas D.K., Miret M., Sørensen H.T., Pedersen L. (2010). Risk of arterial cardiovascular diseases in patients with multiple sclerosis: A population-based cohort study. Neuroepidemiology.

[16] Correale M., Lamacchia O., Ciccarelli M., Dattilo G., Tricarico L., Brunetti N.D. (2021). Vascular and metabolic effects of SGLT2i and GLP-1 in heart failure patients. Heart Fail. Rev..

[17] Gault V.A., Hölscher C. (2018). GLP-1 receptor agonists show neuroprotective effects in animal models of diabetes. Peptides.

[18] Hölscher C. (2014). Central effects of GLP-1: New opportunities for treatments of neurodegenerative diseases. J. Endocrinol..

[19] Kim M.H., Kim E.H., Jung H.S., Yang D., Park E.Y., Jun H.S. (2017). EX4 stabilizes and activates Nrf2 via PKCδ, contributing to the prevention of oxidative stress-induced pancreatic beta cell damage. Toxicol. Appl. Pharmacol..

[20] Breder I., Cunha Breder J., Bonilha I., Munhoz D.B., Medorima S.T.K., Oliveira D.C., do Carmo H.R., Moreira C., Kontush A., Zimetti F. (2020). Rationale and design of the expanded combination of evolocumab plus empagliflozin in diabetes: EXCEED-BHS3 trial. Ther. Adv. Chronic Dis..

[21] Packer M., Anker S.D., Butler J., Filippatos G., Pocock S.J., Carson P., Januzzi J., Verma S., Tsutsui H., Brueckmann M. (2020). Cardiovascular and Renal Outcomes with Empagliflozin in Heart Failure. N. Engl. J. Med..

[22] Nauck M.A., Quast D.R., Wefers J., Pfeiffer A.F.H. (2021). The evolving story of incretins (GIP and GLP-1) in metabolic and cardiovascular disease: A pathophysiological update. Diabetes Obes. Metab..

[23] Nauck M.A., Meier J.J., Cavender M.A., Abd El Aziz M., Drucker D.J. (2017). Cardiovascular Actions and Clinical Outcomes With Glucagon-Like Peptide-1 Receptor Agonists and Dipeptidyl Peptidase-4 Inhibitors. Circulation.

[24] Rizzo M., Chandalia M., Patti A.M., Di Bartolo V., Rizvi A.A., Montalto G., Abate N. (2014). Liraglutide decreases carotid intima-media thickness in patients with type 2 diabetes: 8-month prospective pilot study. Cardiovasc. Diabetol..

[25] Jorsal A., Kistorp C., Holmager P., Tougaard R.S., Nielsen R., Hänselmann A., Nilsson B., Møller J.E., Hjort J., Rasmussen J. (2017). Effect of liraglutide, a glucagon-like peptide-1 analogue, on left ventricular function in stable chronic heart failure patients with and without diabetes (LIVE)-a multicentre, double-blind, randomised, placebo-controlled trial. Eur. J. Heart Fail..

[26] Marso S.P., Holst A.G., Vilsbøll T. (2017). Semaglutide and Cardiovascular Outcomes in Patients with Type 2 Diabetes. N. Engl. J. Med..

[27] Lee Y.S., Jun H.S. (2016). Anti-Inflammatory Effects of GLP-1-Based Therapies beyond Glucose Control. Mediat. Inflamm..

[28] Honigberg M.C., Chang L.S., McGuire D.K., Plutzky J., Aroda V.R., Vaduganathan M. (2020). Use of Glucagon-Like Peptide-1 Receptor Agonists in Patients With Type 2 Diabetes and Cardiovascular Disease: A Review. JAMA Cardiol..

[29] Wei R., Ma S., Wang C., Ke J., Yang J., Li W., Liu Y., Hou W., Feng X., Wang G. (2016). Exenatide exerts direct protective effects on endothelial cells through the AMPK/Akt/eNOS pathway in a GLP-1 receptor-dependent manner. Am. J. Physiol. Endocrinol. Metab..

[30] Feingold K.R., Feingold K.R., Anawalt B., Blackman M.R., Boyce A., Chrousos G., Corpas E., de Herder W.W., Dhatariya K., Dungan K., Hofland J. (2000). Introduction to Lipids and Lipoproteins. Endotext.

[31] Oravec S., Dostal E., Dukát A., Gavorník P., Kucera M., Gruber K. (2011). HDL subfractions analysis: A new laboratory diagnostic assay for patients with cardiovascular diseases and dyslipoproteinemia. Neuroendocrinol. Lett..

[32] Kollar B., Siarnik P., Hluchanova A., Klobucnikova K., Mucska I., Turcani P., Paduchova Z., Katrencikova B., Janubova M., Konarikova K. (2021). The impact of sleep apnea syndrome on the altered lipid metabolism and the redox balance. Lipids Health Dis..

[33] Hluchanova A., Kollar B., Klobucnikova K., Hardonova M., Poddany M., Zitnanova I., Dvorakova M., Konarikova K., Tedla M., Urik M. (2023). Lipoprotein Subfractions Associated with Endothelial Function in Previously Healthy Subjects with Newly Diagnosed Sleep Apnea-A Pilot Study. Life.

[34] Šiarnik P., Čarnická Z., Krivošíková Z., Klobučníková K., Žitňanová I., Kollár B., Sýkora M., Turčáni P. (2017). Association of lipoprotein subfractions with endothelial function and arterial stiffness in acute ischemic stroke. Scand. J. Clin. Lab. Investig..

[35] Margulies K.B., Hernandez A.F., Redfield M.M., Givertz M.M., Oliveira G.H., Cole R., Mann D.L., Whellan D.J., Kiernan M.S., Felker G.M. (2016). Effects of Liraglutide on Clinical Stability Among Patients With Advanced Heart Failure and Reduced Ejection Fraction: A Randomized Clinical Trial. JAMA.

[36] Balestrieri M.L., Rizzo M.R., Barbieri M., Paolisso P., D’Onofrio N., Giovane A., Siniscalchi M., Minicucci F., Sardu C., D’Andrea D. (2015). Sirtuin 6 expression and inflammatory activity in diabetic atherosclerotic plaques: Effects of incretin treatment. Diabetes.

[37] Sposito A.C., Berwanger O., de Carvalho L.S.F., Saraiva J.F.K. (2018). GLP-1RAs in type 2 diabetes: Mechanisms that underlie cardiovascular effects and overview of cardiovascular outcome data. Cardiovasc. Diabetol..

[38] Oh Y.S., Jun H.S. (2017). Effects of Glucagon-Like Peptide-1 on Oxidative Stress and Nrf2 Signaling. Int. J. Mol. Sci..

[39] Silva-Palacios A., Königsberg M., Zazueta C. (2016). Nrf2 signaling and redox homeostasis in the aging heart: A potential target to prevent cardiovascular diseases?. Ageing Res. Rev..

[40] Li J., Ichikawa T., Villacorta L., Janicki J.S., Brower G.L., Yamamoto M., Cui T. (2009). Nrf2 protects against maladaptive cardiac responses to hemodynamic stress. Arterioscler. Thromb. Vasc. Biol..

[41] Laviola L., Leonardini A., Melchiorre M., Orlando M.R., Peschechera A., Bortone A., Paparella D., Natalicchio A., Perrini S., Giorgino F. (2012). Glucagon-like peptide-1 counteracts oxidative stress-dependent apoptosis of human cardiac progenitor cells by inhibiting the activation of the c-Jun N-terminal protein kinase signaling pathway. Endocrinology.

[42] Axtell A.L., Gomari F.A., Cooke J.P. (2010). Assessing endothelial vasodilator function with the Endo-PAT 2000. J. Vis. Exp..

[43] Hoefner D.M., Hodel S.D., O’Brien J.F., Branum E.L., Sun D., Meissner I., McConnell J.P. (2001). Development of a rapid, quantitative method for LDL subfractionation with use of the Quantimetrix Lipoprint LDL System. Clin. Chem..

[44] Re R., Pellegrini N., Proteggente A., Pannala A., Yang M., Rice-Evans C. (1999). Antioxidant activity applying an improved ABTS radical cation decolorization assay. Free Radic. Biol. Med..

